# Stapler vs. sleeve circumcision for multiple penile condylomata acuminata: a comparative study

**DOI:** 10.3389/fsurg.2026.1846470

**Published:** 2026-08-20

**Authors:** Haoran Chen, Yangfeng Lou, Longfei Yang, Peng Zhou

**Affiliations:** 1 Department of General Surgery, Hangzhou Third People’s Hospital, Hangzhou, China; 2 Department of Urology, Hangzhou Third People’s Hospital, Hangzhou, China

**Keywords:** circumcision, condylomata acuminata, cosmetic outcome, disposable circumcision stapler, human papillomavirus, penile warts, recurrence, stapler circumcision

## Abstract

**Background:**

Multiple penile condylomata acuminata (CA) often involve the inner foreskin and frenulum. This retrospective study compared conventional sleeve and disposable circular stapler circumcision, each combined with ablation of visible lesions, in selected patients with multiple penile CA.

**Methods:**

Consecutive patients aged 18–55 years with at least 5 visible lesions involving the foreskin and/or frenulum who underwent treatment between January 2020 and December 2024 were screened. Propensity-score matching (1:1 nearest neighbor, caliper 0.05) using prespecified covariates yielded 41 patients per group. Outcomes included operative time, blood loss, complications, baseline and 12-month IIEF-15, cosmetic appearance, patient satisfaction, and 12-month recurrence.

**Results:**

After matching, baseline characteristics were well balanced (all standardized mean differences <0.1). Compared with sleeve circumcision, stapler circumcision was associated with shorter operative time (mean difference −14.2 min, 95% CI −16.7 to −11.7), less blood loss (mean difference −7.5 mL, 95% CI −9.2 to −5.8), higher patient- and surgeon-rated cosmetic scores (mean differences 0.7 and 0.8, respectively), and higher patient satisfaction (mean difference 0.7). IIEF-15 scores were similar. Recurrence occurred in 6/41 (14.6%) stapler patients and 8/41 (19.5%) sleeve patients (risk difference −4.9%, 95% CI −21.1% to 11.4%; odds ratio 0.71, 95% CI 0.22 to 2.26). No major complications occurred.

**Conclusion:**

In this single-center retrospective matched cohort of carefully selected patients without anatomical contraindications to stapler placement, stapler circumcision was associated with perioperative and patient-reported advantages. The recurrence estimate was imprecise and does not establish equivalence or non-inferiority. These findings are hypothesis-generating and require confirmation in larger prospective studies with longer follow-up.

## Introduction

Condylomata acuminata (CA), or anogenital warts, is a common sexually transmitted infection in men. Current international guidelines emphasize that approximately 90% of anogenital warts are attributable to low-risk human papillomavirus (HPV) types 6 and 11, although oncogenic HPV types may occasionally be detected as co-infections (1–3). In uncircumcised men, lesions commonly occur under the foreskin and may involve the inner prepuce, frenulum, coronal sulcus, and adjacent penile skin; moisture, friction, and subclinical HPV persistence may contribute to recurrence.

Treatment selection for anogenital warts should be individualized according to lesion size, number, and anatomical site, as well as patient preference, treatment cost, adverse effects, treatment availability, and clinician experience (1–3). For extensive or anatomically difficult lesions, provider-administered ablative or surgical modalities, including electrosurgery, CO2 laser, and surgical removal, are recognized options in contemporary CDC, IUSTI-Europe, and BASHH guidance (1–3). In patients with multiple penile CA involving the foreskin and/or frenulum, circumcision combined with ablation of visible lesions is therefore a clinically relevant strategy to remove the preputial reservoir and treat concomitant wart burden.

Conventional sleeve circumcision and disposable circular stapler circumcision are two commonly used surgical techniques. Previous studies and systematic reviews in general circumcision populations have reported shorter operative time, reduced bleeding, and favorable cosmetic outcomes with stapler or device-assisted approaches (4–7). However, comparative evidence specifically focused on adults with multiple penile CA remains limited. This single-center retrospective comparative study used propensity-score matching to compare perioperative, functional, cosmetic, patient-reported, and 12-month recurrence outcomes between disposable circular stapler circumcision and conventional sleeve circumcision in this population.

## Materials and methods

### Study design and reporting

**Figure 1:**
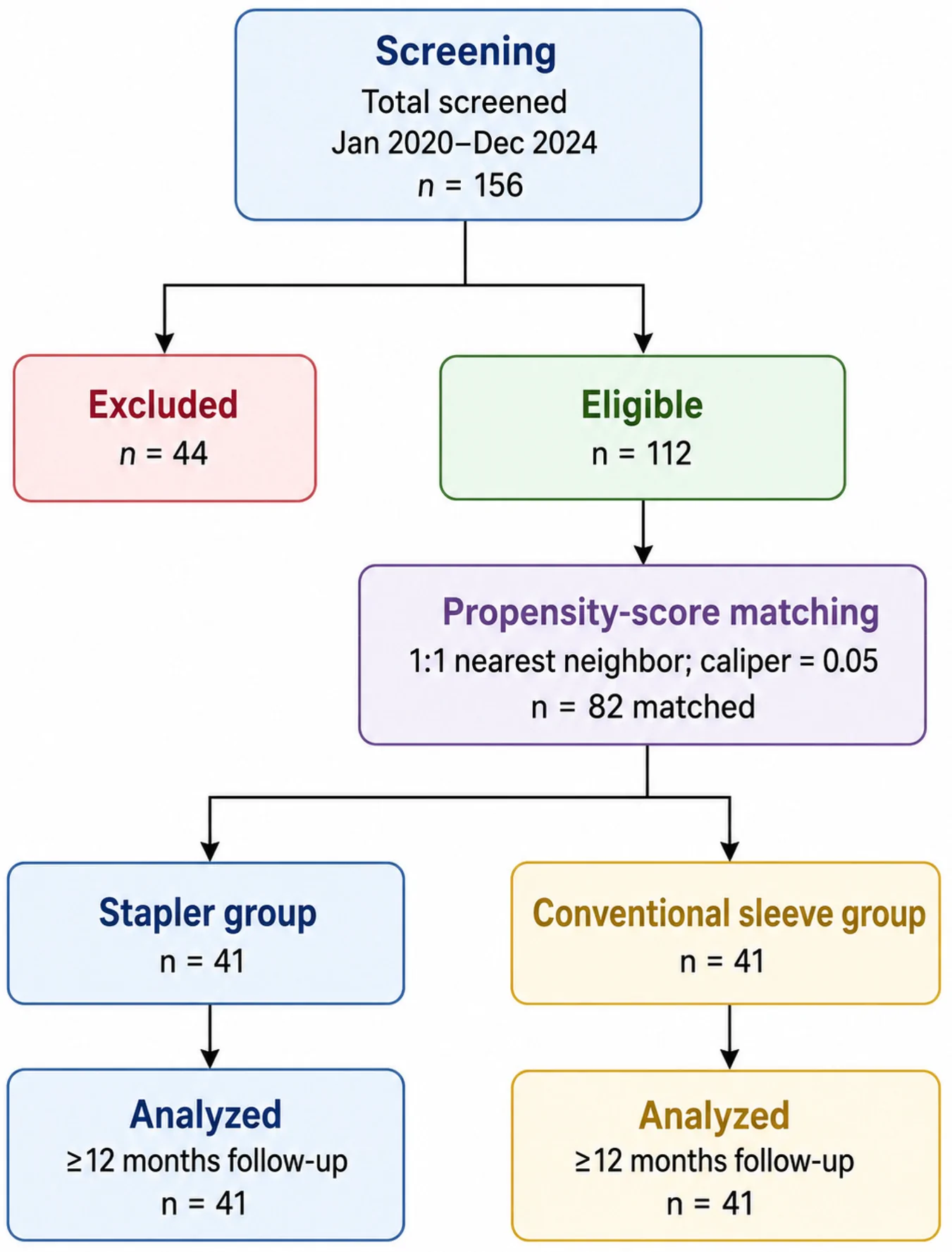
Flowchart of patient screening, eligibility assessment, and propensity-score matching.

This single-center retrospective observational study was approved by the Ethics Committee of Hangzhou Third People's Hospital (Approval No. 2025-KA300) and was reported according to the STROBE statement. All consecutive patients identified from the hospital electronic medical record system using ICD-10 code A63.0 and procedure codes for circumcision between January 2020 and December 2024 were screened. No sampling was performed after electronic identification. A completed STROBE checklist is provided as Supplementary Table S1, and a directed acyclic graph describing the assumed treatment-selection and outcome pathways is provided as Supplementary Figure S1. The patient screening, eligibility assessment, and propensity-score matching process is summarized in Figure 1.

### Eligibility criteria

Patients were eligible if they were 18–55 years old, had clinically diagnosed multiple penile CA (at least 5 visible lesions, confirmed by acetowhite testing and/or dermoscopy), had at least partial involvement of the inner foreskin and/or frenulum, underwent circumcision with concomitant ablation of visible lesions, and completed at least 12 months of follow-up. Patients were excluded for immunodeficiency or ongoing immunosuppressive therapy, uncontrolled diabetes, suspected penile malignancy, previous circumcision, severe phimosis or severe frenular shortening unsuitable for safe stapler placement, local destructive or antiviral therapy within the preceding 3 months, extensive glans/coronal sulcus/meatal disease requiring a more complex procedure, or incomplete follow-up. Accordingly, the cohort represents carefully selected patients without anatomical contraindications to stapler placement, and the findings should not be extrapolated to patients with the excluded anatomical or clinical conditions.

### Variables considered for confounding control

Variables were selected for propensity-score matching based on clinical relevance and an *a priori* causal framework: age, lesion burden (≥10 vs. < 10 visible lesions), foreskin involvement extent, frenular involvement, high-risk HPV co-infection status/missingness, concomitant ablation method, and smoking status. Lesion distribution (frenular-predominant vs. inner-preputial-predominant), previous topical/destructive therapy before the 3-month exclusion window, sexual behavior, partner HPV status, device availability, and surgeon/patient preference were considered potential residual or unmeasured confounders and are addressed in the sensitivity analysis and limitations.

### Stapler technique

**Figure 2:**
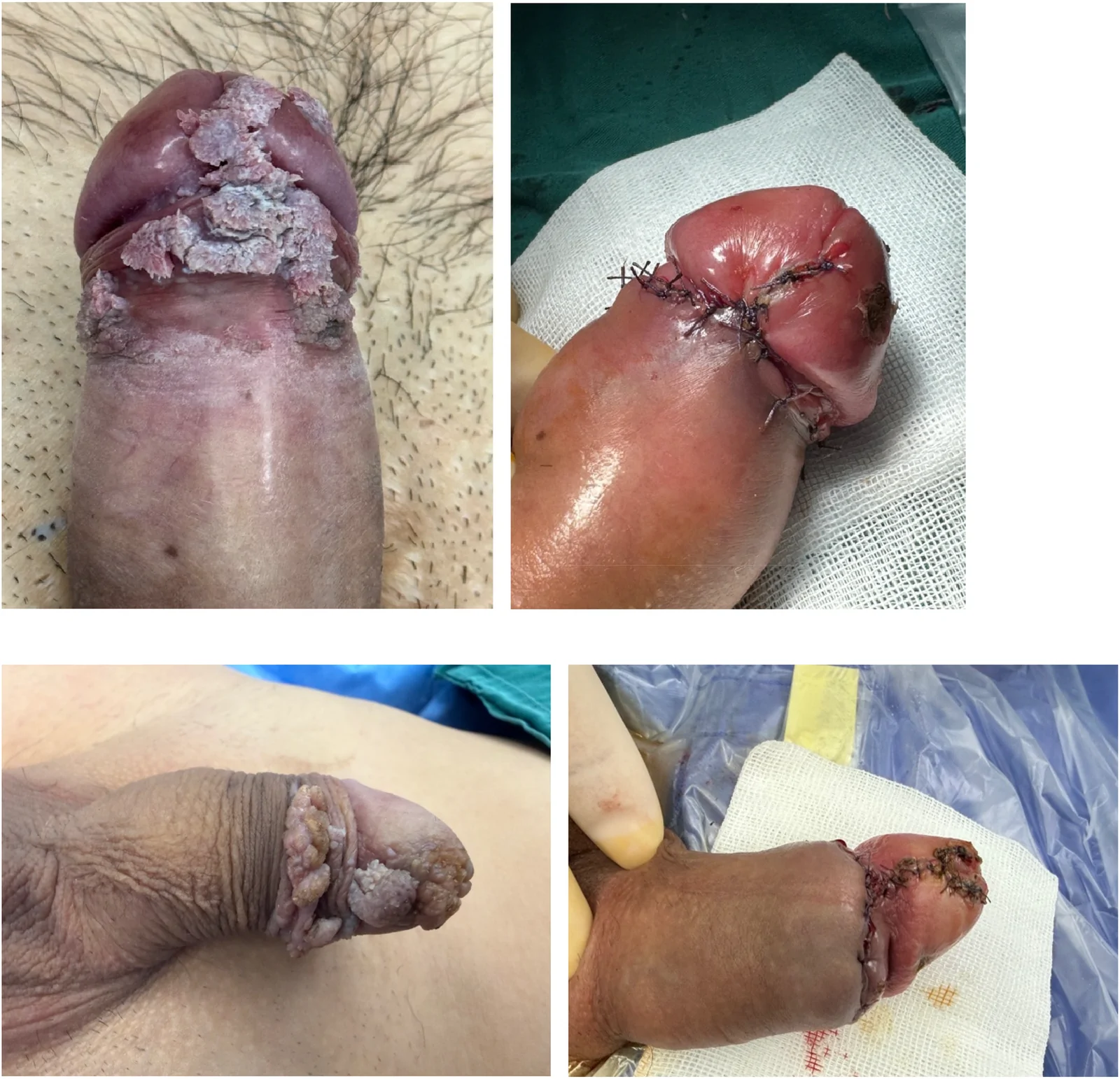
Representative preoperative and postoperative images of conventional sleeve circumcision for multiple penile condylomata acuminata.

**Figure 3:**
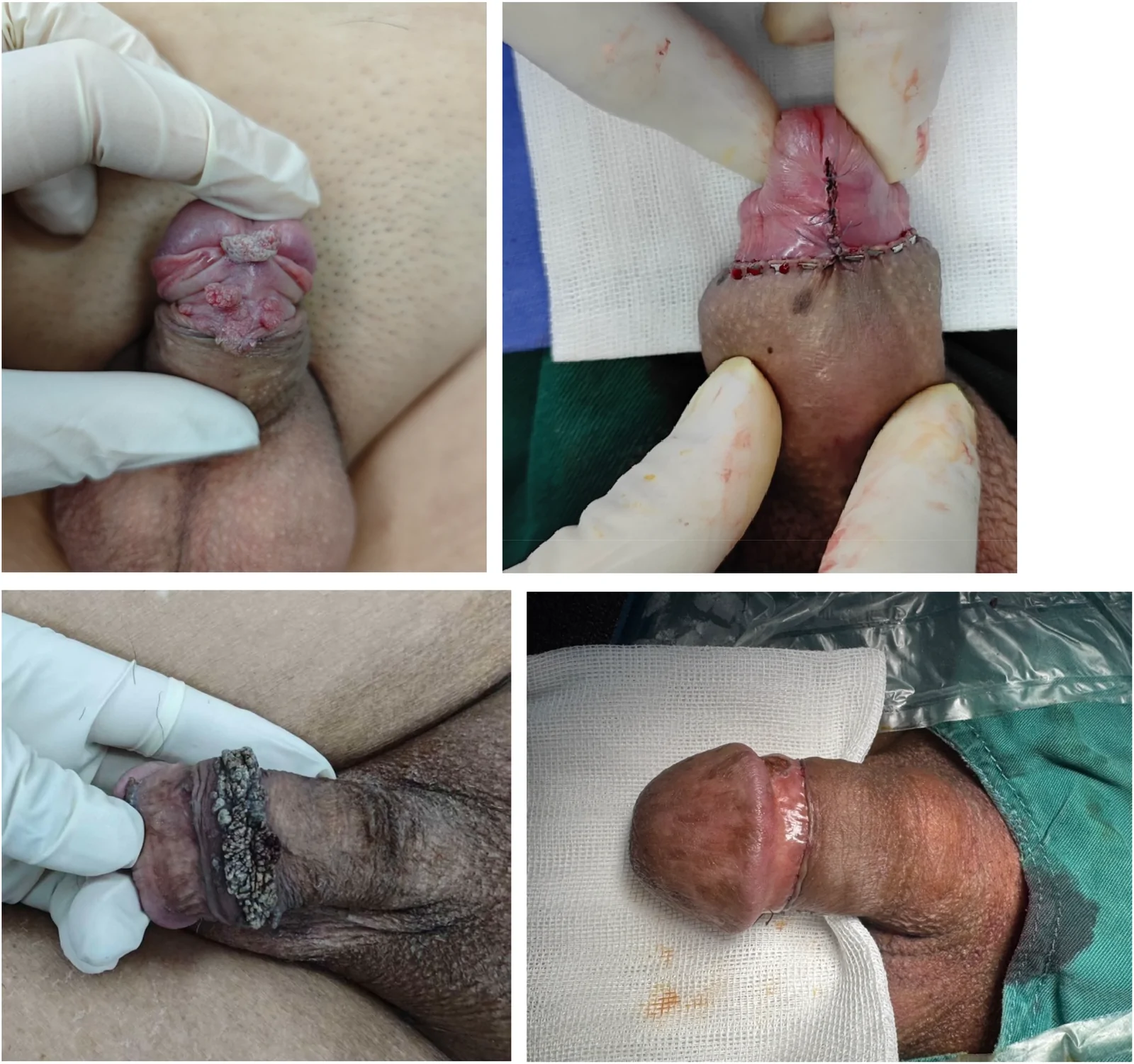
Representative preoperative and postoperative images of disposable circular stapler circumcision for multiple penile condylomata acuminata.

Disposable circular staplers (Jiangxi Langhe Medical Treatment Appliance Technology Co., Ltd.; 26, 28, or 30 mm) were selected according to glans diameter and preputial circumference after intraoperative exposure. The smallest size that allowed complete bell coverage of the glans without compression was used. After dorsal penile block or local anesthesia and standard sterile preparation, the bell was positioned beneath the foreskin with the frenulum aligned ventrally. The foreskin was drawn evenly over the bell to avoid asymmetric resection, the device was tightened, compression was maintained before firing, and the staple line was inspected after firing. Additional hemostasis was performed only if focal oozing was observed. Visible CA lesions involving the frenulum were ablated or trimmed under direct vision; severe frenular shortening unsuitable for stapler placement was an exclusion criterion. No intraoperative conversion from stapler to sleeve circumcision was recorded. Representative preoperative and postoperative appearances after conventional sleeve and disposable circular stapler circumcision are shown in Figures 2, 3, respectively.

### Outcome assessment and blinding

IIEF-15 was assessed preoperatively and at 12 months using the validated Chinese-language questionnaire available in routine practice. Cosmetic appearance and patient satisfaction were recorded at 12 months using 0-10 visual analogue scales. The surgeon-rated cosmetic score was assigned by a urologist not involved in the index operation. However, neither patients nor the surgeon rater could be reliably blinded to surgical technique because the circumferential staple-line scar was often recognizable. Patient expectations and observer awareness may therefore have influenced the cosmetic and satisfaction ratings; these subjective outcomes were interpreted as exploratory.

### Statistical analysis

Continuous outcomes are reported as mean ± SD or median (IQR), as appropriate, and between-group differences are presented with 95% confidence intervals. Categorical variables were compared using chi-square tests or Fisher exact tests when expected cell counts were small. Propensity scores were estimated by logistic regression and matched 1:1 without replacement using nearest-neighbor matching with a caliper of 0.05. Covariate balance was assessed using standardized mean differences, with <0.1 considered adequate. Recurrence was analyzed as an exploratory endpoint using risk difference, risk ratio, and odds ratio with 95% confidence intervals. Because only 14 recurrence events occurred, a full multivariable logistic regression model was not reported to avoid overfitting. A descriptive E-value was calculated for the recurrence risk-ratio point estimate. No *a priori* sample-size calculation was performed because this retrospective study included all available eligible cases. After the cohort was fixed, a *post hoc* calculation was used only to contextualize the precision of the recurrence analysis: with 41 patients per group, a two-sided alpha of 0.05, and the observed absolute difference of −4.9 percentage points, power was approximately 9%. This calculation was not used as evidence of equivalence. Statistical analyses were performed using SPSS 26.0 and R 4.3.0.

## Results

Baseline characteristics before and after propensity-score matching are summarized in Table 1. After matching, all prespecified covariates had standardized mean differences below 0.1.

**Table 1:** Baseline patient characteristics before and after propensity-score matching.

Characteristic	Before matching: Stapler (*n* = 60)	Before matching: Sleeve (*n* = 52)	SMD	After matching: Stapler (*n* = 41)	After matching: Sleeve (*n* = 41)	SMD
Age (years), mean ± SD	31.8 ± 4.7	34.2 ± 8.1	0.31	32.5 ± 7.2	33.1 ± 7.5	0.08
Number of visible lesions, median (IQR)	12 (8–18)	9 (6–14)	0.38	10 (7–15)	10 (7–16)	0.06
Lesions ≥10, *n* (%)	36 (60.0)	21 (40.4)	0.40	23 (56.1)	22 (53.7)	0.05
Extensive foreskin involvement^a^, *n* (%)	48 (80.0)	35 (67.3)	0.28	32 (78.0)	31 (75.6)	0.06
Frenular involvement, *n* (%)	42 (70.0)	31 (59.6)	0.22	27 (65.9)	28 (68.3)	0.05
High-risk HPV co-infection^b^, *n* (%)	12 (20.0)	8 (15.4)	0.13	7 (17.1)	8 (19.5)	0.06
Ablation method: electrocautery, *n* (%)	42 (70.0)	28 (53.8)	0.34	25 (61.0)	24 (58.5)	0.05
Current smoker, *n* (%)	18 (30.0)	20 (38.5)	0.18	14 (34.1)	15 (36.6)	0.05

SMD, standardized mean difference; all post-matching SMD values were <0.1.

a Extensive foreskin involvement was defined as >50% of the inner foreskin surface affected.

b High-risk HPV co-infection was based on genotyping in 78/82 matched patients, with four missing results evenly distributed.

### Primary outcomes with confidence intervals

Stapler circumcision was associated with shorter operative time (18.4 ± 4.2 vs. 32.6 ± 6.8 min; mean difference −14.2 min, 95% CI −16.7 to −11.7; *P* < 0.001) and lower blood loss (6.8 ± 2.5 vs. 14.3 ± 4.9 mL; mean difference −7.5 mL, 95% CI −9.2 to −5.8; *P* < 0.001). Patient-rated cosmetic scores (9.1 ± 0.7 vs. 8.4 ± 0.9; mean difference 0.7, 95% CI 0.35 to 1.05; *P* < 0.001), surgeon-rated cosmetic scores (9.3 ± 0.6 vs. 8.5 ± 0.8; mean difference 0.8, 95% CI 0.49 to 1.11; *P* < 0.001), and patient satisfaction (9.3 ± 0.6 vs. 8.6 ± 0.8; mean difference 0.7, 95% CI 0.39 to 1.01; *P* < 0.001) favored stapler circumcision. IIEF-15 scores were similar between groups at baseline (68.7 ± 4.6 vs. 68.2 ± 5.0; mean difference 0.5, 95% CI −1.6 to 2.6; *P* = 0.641) and at 12 months (68.9 ± 4.3 vs. 67.8 ± 4.7; mean difference 1.1, 95% CI −0.88 to 3.08; *P* = 0.272). Perioperative and 12-month outcomes are summarized in Table 2.

**Table 2:** Perioperative and 12-month outcomes between the groups.

Parameter	Stapler (*n* = 41)	Sleeve (*n* = 41)	Effect estimate (95% CI)	*P* value
Operative time (min), mean ± SD	18.4 ± 4.2	32.6 ± 6.8	−14.2 (−16.7 to −11.7)	<0.001
Blood loss (mL), mean ± SD	6.8 ± 2.5	14.3 ± 4.9	−7.5 (−9.2 to −5.8)	<0.001
IIEF-15 baseline, mean ± SD	68.7 ± 4.6	68.2 ± 5.0	0.5 (−1.6 to 2.6)	0.641
IIEF-15 at 12 mo, mean ± SD	68.9 ± 4.3	67.8 ± 4.7	1.1 (−0.88 to 3.08)	0.272
Patient cosmetic score at 12 mo, mean ± SD	9.1 ± 0.7	8.4 ± 0.9	0.7 (0.35 to 1.05)	<0.001
Surgeon cosmetic score at 12 mo, mean ± SD	9.3 ± 0.6	8.5 ± 0.8	0.8 (0.49 to 1.11)	<0.001
Patient satisfaction at 12 mo, mean ± SD	9.3 ± 0.6	8.6 ± 0.8	0.7 (0.39 to 1.01)	<0.001
Any minor postoperative event, *n* (%)	4 (9.8)	12 (29.3)	OR 0.26 (0.08 to 0.90)	0.049
Recurrence at 12 mo, *n* (%)	6 (14.6)	8 (19.5)	RD −4.9% (−21.1 to 11.4); OR 0.71 (0.22 to 2.26)	0.770
Direct procedural cost (USD), mean ± SD	336.42 ± 7.30	145.67 ± 8.42	190.75 (187.29 to 194.21)	<0.001

Fisher exact tests were used for sparse categorical outcomes. Mean differences are shown for continuous outcomes; negative values favor stapler circumcision for time and blood loss. Direct procedural cost is reported descriptively and is not a cost-effectiveness estimate.

### Recurrence and HPV genotyping

Twelve-month recurrence occurred in 6/41 (14.6%) patients in the stapler group and 8/41 (19.5%) patients in the sleeve group (risk difference −4.9%, 95% CI −21.1% to 11.4%; risk ratio 0.75, 95% CI 0.29 to 1.97; odds ratio 0.71, 95% CI 0.22 to 2.26; Fisher exact *P* = 0.770). HPV genotyping was available in 78/82 patients. Low-risk HPV types 6/11 predominated. High-risk HPV co-infection was not statistically associated with recurrence in univariable analysis (unadjusted odds ratio 1.93, 95% CI 0.51−7.28; Fisher exact *P* = 0.453). Sensitivity analyses treating the four missing genotyping results as low-risk only or as high-risk co-infection did not materially alter the treatment-outcome comparisons. The E-value for the recurrence risk-ratio point estimate was 2.00, but the confidence interval crossed the null; therefore, this analysis was used only to illustrate sensitivity to unmeasured confounding and does not support a causal recurrence claim. The HPV genotyping distribution and recurrence according to HPV risk group are summarized in Table 3.

**Table 3:** HPV genotyping distribution and relation to recurrence between the groups.

Parameter	Stapler (*n* = 38)	Sleeve (*n* = 40)	Total (*n* = 78)	*P* value
Low risk only (6/11 ± 42/43/44)	31 (81.6%)	32 (80.0%)	63 (80.8%)	1.00
High-risk co-infection (any HR type)	7 (18.4%)	8 (20.0%)	15 (19.2%)	1.00
– HPV 16	2	3	5	
– HPV 18	1	1	2	
– HPV 52/58	3	3	6	
– Others (31/33/51)	1	1	2	
Recurrence in low-risk only subgroup	4/31 (12.9%)	6/32 (18.8%)	10/63 (15.9%)	0.73
Recurrence in high-risk co-infection	2/7 (28.8%)	2/8 (25.0%)	4/15 (26.7%)	1.00

### Safety and procedural details

No major complications occurred. Overall minor postoperative events were less frequent after stapler circumcision than after sleeve circumcision (4/41 [9.8%] vs. 12/41 [29.3%]; odds ratio 0.26, 95% CI 0.08 to 0.90; Fisher exact *P* = 0.049). Individual minor events were uncommon and should be interpreted descriptively because of sparse counts. Staples detached spontaneously within 14-21 days. No intraoperative conversion from stapler to sleeve circumcision was recorded. Detailed minor postoperative events are presented in Table 4.

**Table 4:** Detailed minor postoperative events.

Parameter	Stapler (*n* = 41)	Sleeve (*n* = 41)	Effect estimate (95% CI)	*P* value
Pain score on postoperative day 1 (VAS 0–10), mean ± SD	4.2 ± 1.1	4.5 ± 1.3	−0.3 (−0.83 to 0.23)	0.263
Minor bleeding/oozing not requiring intervention, *n* (%)	2 (4.9)	3 (7.3)	OR 0.65 (0.10 to 4.10)	1.000
Wound dehiscence, *n* (%)	0 (0)	4 (9.8)	Not estimable	0.116
Severe edema, *n* (%)	1 (2.4)	3 (7.3)	OR 0.32 (0.03 to 3.18)	0.616
Minor wound infection, *n* (%)	1 (2.4)	2 (4.9)	OR 0.49 (0.04 to 5.57)	1.000
Granuloma formation, *n* (%)	0 (0)	1 (2.4)	Not estimable	1.000
Other minor events (mild bruising), *n* (%)	2 (4.9)	3 (7.3)	OR 0.65 (0.10 to 4.10)	1.000
Time to return to work (days), mean ± SD	10.5 ± 2.3	12.1 ± 2.7	−1.6 (−2.70 to −0.50)	0.005

Fisher exact tests were used for categorical variables and *t/*Welch tests for continuous variables. Individual minor-event analyses are descriptive because of sparse counts.

### Direct procedural costs

The direct procedural cost was higher in the stapler group ($336.42 ± 7.30 vs. $145.67 ± 8.42; mean difference $190.75, 95% CI $187.29 to $194.21; *P* < 0.001). Only direct procedural costs were available. Anesthesia, operating-room overhead, recovery time, patient productivity, health utilities, and willingness-to-pay thresholds were not collected; therefore, neither cost-effectiveness nor an incremental cost per operative minute saved can be estimated. The cost comparison is descriptive only.

## Discussion

In this retrospective propensity-score-matched cohort of Chinese men with multiple penile condylomata acuminata, disposable circular stapler circumcision was associated with shorter operative time, less intraoperative blood loss, fewer overall minor postoperative events, and higher patient-reported cosmetic and satisfaction scores than conventional sleeve circumcision. Baseline and 12-month IIEF-15 scores were similar between groups. The recurrence estimate was imprecise, and no inference of equivalence, non-inferiority, or absence of clinically important difference is supported. These associations should be regarded as hypothesis-generating.

The perioperative advantages observed in the stapler group are biologically and technically plausible. The disposable circular stapler allows simultaneous cutting and stapling, producing a uniform circumferential wound edge and immediate mechanical hemostasis. This may reduce operative time, blood loss, and wound-edge irregularity compared with conventional sleeve circumcision, which requires manual dissection and suturing. These findings are broadly consistent with previous reports and systematic reviews of device-assisted circumcision in non-CA populations (4–7). Nevertheless, direct comparative evidence in adult patients with multiple penile CA remains limited, and extrapolation from general circumcision populations should be made cautiously.

The recurrence findings require a conservative interpretation. Although the observed 12-month recurrence rate was numerically lower in the stapler group, the risk-difference confidence interval (−21.1% to 11.4%) encompassed both a clinically important decrease and a clinically important increase. With only 14 recurrence events and approximately 9% *post hoc* power to detect the observed difference, the study cannot determine whether recurrence differs between the techniques and does not establish equivalence or non-inferiority. Previous evidence suggests that circumcision may reduce penile HPV prevalence, acquisition, or persistence and may increase site-specific HPV clearance in some settings (8–11). However, recurrence of HPV-related disease may occur beyond 12 months, particularly in patients with persistent HPV exposure, partner reinfection, or subclinical lesions. Longer follow-up is needed before firm conclusions can be drawn regarding recurrence control.

High-risk HPV co-infection was not statistically associated with recurrence in this cohort, but the number of high-risk HPV-positive patients was small and genotyping was incomplete in four matched patients. Low-risk HPV types 6/11 predominated, which is consistent with international guideline statements and epidemiological evidence indicating that HPV 6/11 account for most anogenital warts (1–3, 12). The HPV subgroup findings should therefore be considered descriptive rather than definitive.

Persistent infection with oncogenic HPV is an established etiologic pathway for penile squamous cell carcinoma, while phimosis, chronic inflammation, smoking, and poor hygiene are additional risk factors. Circumcision may reduce some of these risk pathways by improving hygiene and potentially reducing HPV acquisition or persistence, but it does not eliminate penile cancer risk. Because the present study excluded suspected malignancy, predominantly enrolled patients with low-risk HPV 6/11, and followed patients for only 12 months, it cannot address penile cancer prevention (13). More broadly, HPV infection and HPV-related diseases remain a substantial global health burden (14).

From a clinical perspective, stapler circumcision may be a practical option for carefully selected patients with multiple penile CA involving the foreskin and/or frenulum. Treatment choice should incorporate lesion characteristics, anatomic location, patient preference, cost, adverse effects, treatment availability, and provider experience (1–3). The present findings apply only to patients without anatomical contraindications to stapler placement and should not be extrapolated to patients with severe phimosis, severe frenular shortening, extensive glans/coronal sulcus/meatal disease, or other excluded conditions; conventional sleeve circumcision or an individualized surgical approach may be more appropriate in such cases.

The disposable stapler was associated with approximately $191 higher direct procedural cost per case. This isolated difference cannot determine economic value because anesthesia, operating-room overhead, recovery time, patient productivity, health utilities, and willingness-to-pay thresholds were not measured. The cost data are descriptive and should not be interpreted as a cost-effectiveness analysis or as an incremental cost per operative minute saved.

This study has several limitations. First, its retrospective observational design remains vulnerable to residual confounding despite propensity-score matching. Important factors such as detailed lesion micro-distribution, prior therapies outside the exclusion window, sexual behavior, partner HPV status, device availability, and surgeon or patient preference were incompletely captured. Second, the matched sample was modest, with only 41 patients per group and 14 recurrence events; recurrence and rare-complication analyses were therefore underpowered and exploratory. Third, the extensive eligibility restrictions and the single-center Chinese cohort aged 18-55 years limit generalizability, particularly to patients with severe phimosis, severe frenular shortening, extensive glans/coronal sulcus/meatal disease, suspected malignancy, immunodeficiency, or other excluded conditions. Fourth, follow-up was limited to 12 months, so late HPV-related recurrence may have been missed. Fifth, neither patients nor the surgeon rater could be fully blinded because the stapler scar pattern was identifiable. Patient expectations and observer awareness could therefore have biased both patient- and surgeon-rated cosmetic and satisfaction outcomes; these associations should not be interpreted as objective cosmetic superiority. Sixth, cost data were limited to direct procedural costs and did not include a formal economic evaluation. Accordingly, all comparative findings are hypothesis-generating. Future multicenter prospective or randomized studies should include longer follow-up, blinded photographic assessment where feasible, standardized sexual-function assessment from baseline, and formal health-economic analysis.

## Conclusion

In carefully selected patients with multiple penile CA and no anatomical contraindication to stapler placement, stapler circumcision was associated with shorter operative time, less blood loss, and higher subjective cosmetic and satisfaction scores. This study was not powered to determine whether 12-month recurrence differs between techniques, and the wide confidence interval includes clinically important benefit and harm. The findings are hypothesis-generating and do not support equivalence, non-inferiority, or a claim that stapler circumcision does not increase recurrence. Larger multicenter prospective studies with longer follow-up and blinded outcome assessment are required.

## Data Availability

The raw data supporting the conclusions of this article will be made available by the authors, without undue reservation.
