# Supplementary material for: Stapler vs. sleeve circumcision for multiple penile condylomata acuminata: a comparative study
Source: Front Surg. 2026 Aug 20;13:1846470. doi: 10.3389/fsurg.2026.1846470 (PMC13538843; doi:10.3389/fsurg.2026.1846470)
Supplement: Supplementary file 1 [file Supplementaryfile1.docx]

**Supplementary Table S1. STROBE checklist for observational studies**

Manuscript: Stapler vs Sleeve Circumcision for Multiple Penile Condylomata Acuminata: A Comparative Study

Study design: single-center retrospective observational matched cohort study.

| **Item** | **STROBE domain** | **Recommendation** | **Where addressed in manuscript** |
| --- | --- | --- | --- |
| 1 | Title and abstract | Indicate the study design in the title or abstract and provide an informative summary. | Title/Abstract: retrospective comparative matched cohort, PSM, outcomes, limitations. |
| 2 | Background/rationale | Explain the scientific background and rationale. | Introduction: burden of anogenital warts, guideline-based management, evidence gap for multiple penile CA. |
| 3 | Objectives | State specific objectives, including any prespecified hypotheses. | End of Introduction: comparison of perioperative, functional, cosmetic, patient-reported, and recurrence outcomes. |
| 4 | Study design | Present key elements of study design early. | Methods, Study design and reporting: single-center retrospective observational study. |
| 5 | Setting | Describe setting, locations, and relevant dates. | Methods: Hangzhou Third People’s Hospital; January 2020 to December 2024. |
| 6 | Participants | Describe eligibility criteria, sources, and selection methods. | Methods, Eligibility criteria; Figure 1 flowchart. |
| 7 | Variables | Define outcomes, exposures, predictors, confounders, and effect modifiers. | Methods: outcomes, surgical technique, variables considered for confounding control; Supplementary Table S2. |
| 8 | Data sources/measurement | Describe sources of data and methods of assessment. | Methods: EMR identification, procedure codes, HPV genotyping, IIEF-15, VAS assessment, follow-up visits. |
| 9 | Bias | Describe efforts to address potential sources of bias. | Methods: PSM, SMD balance, DAG, blinding limitations; Discussion limitations. |
| 10 | Study size | Explain how study size was arrived at. | Results, Power and precision: retrospective eligible sample; post-hoc power for recurrence. |
| 11 | Quantitative variables | Explain handling of quantitative variables. | Methods: continuous variables as mean ± SD or median (IQR); lesion burden dichotomized as ≥10 vs. <10. |
| 12 | Statistical methods | Describe statistical methods, confounding control, subgroup/sensitivity analyses, and missing data handling. | Methods/Results: PSM, Fisher exact tests, 95% CIs, recurrence RD/RR/OR, E-value, HPV missingness sensitivity. |
| 13 | Participants | Report numbers at each stage and reasons for exclusion. | Figure 1 flowchart; Methods and Results. |
| 14 | Descriptive data | Give characteristics of study participants and missing data. | Table 1 baseline characteristics; Table 3 HPV genotyping availability. |
| 15 | Outcome data | Report numbers of outcome events or summary measures. | Tables 2–4; Results: recurrence, complications, costs, IIEF-15, cosmetic/satisfaction scores. |
| 16 | Main results | Give unadjusted/adjusted estimates and precision; indicate confounder adjustment. | Results: mean differences, RD/RR/OR and 95% CIs; PSM balance; no full multivariable model due sparse events. |
| 17 | Other analyses | Report subgroup/sensitivity analyses. | Results: HPV missingness sensitivity; descriptive E-value for recurrence. |
| 18 | Key results | Summarize key results with reference to objectives. | Discussion first paragraph and Conclusion. |
| 19 | Limitations | Discuss limitations, direction and magnitude of potential bias. | Discussion limitations: retrospective design, residual confounding, small sample, short follow-up, non-blinding, cost limitations. |
| 20 | Interpretation | Give cautious overall interpretation considering objectives, limitations, and evidence. | Discussion and Conclusion: hypothesis-generating interpretation; no recurrence equivalence claim. |
| 21 | Generalizability | Discuss external validity. | Discussion limitations: single Chinese center, age range 18–55, HPV distribution, device/cost setting. |
| 22 | Funding | Give funding source and role of funders. | Funding: no funding source. |

Note: This checklist is provided to support transparent reporting and does not replace editorial or statistical review.
